# 1.5 Tesla Magnetic Resonance Imaging Features of Canine Intracranial Intra-axial Hematomas

**DOI:** 10.3389/fvets.2021.778320

**Published:** 2021-12-24

**Authors:** James Whitlock, Andrew Holdsworth, Carles Morales, Laurent Garosi, Inés Carrera

**Affiliations:** ^1^Willows Referral Centre, Part of Linnaeus Veterinary Limited, Solihull, United Kingdom; ^2^Davies Veterinary Specialists, Hitchin, United Kingdom; ^3^Vet Oracle, Norfolk, United Kingdom; ^4^Ars Veterinary Hospital, Barcelona, Spain

**Keywords:** MRI, dog, hemorrhage, intracranial, hematoma

## Abstract

The differentiation of solitary intra-axial hematomas from hemorrhagic neoplasms based on their magnetic resonance imaging (MRI) features is challenging. The treatment and prognosis for these two disease entities are vastly different and distinction between them is often based on MRI findings alone. The aim of this study was to describe the 1.5 tesla MRI features of canine intra-axial hematomas and correlate these findings with the evolution of hemorrhages described in human brains. Retrospective evaluation of patient details, clinical signs, and MRI findings of dogs with intra-axial hematomas that were histopathologically confirmed or determined via repeat MRI study and/or resolution of neurological signs. Ten dogs met the inclusion criteria. All 10 hematoma lesions were determined to be 2–7 days in age. On MRI, all 10 hemorrhagic lesions were comprised of two distinct regions; a relatively thin T1-weighted (T1W), T2-weighted (T2W) and gradient echo (GRE) hypointense (9/10) peripheral border region and a large central region that was heterogenous but predominantly T1W, T2W and GRE hyperintense (8/10). The peripheral border region was complete in its integrity in all 10 cases on T2W and GRE sequences. Contrast enhancement was present in (6/10) hematoma lesions and was always peripheral in nature with no evidence of central enhancement associated with any of the lesions. An intra-axial hematoma should be suspected in solitary hemorrhagic space occupying lesions that have a complete hypointense peripheral rim, elicit a peripheral contrast enhancement pattern, and display the expected temporal pattern of hematoma evolution.

## Introduction

Intracranial hemorrhage is detected accurately with magnetic resonance imaging (MRI) in both human and veterinary medicine (1–3). The simultaneous evaluation of T1-weighted (T1W), T2-weighted (T2W), fluid-attenuated inversion recovery (FLAIR), T2^*^-weighted gradient echo (T2^*^) and/or susceptibility-weighted imaging (SWI) sequences of the brain is highly sensitive and specific for the identification of intracranial hemorrhage (4–6). Consequently, MRI is the modality of choice for the detection of intracranial hemorrhage (2).

The MRI appearance of intracranial hemorrhage can be complex and has different signal intensity patterns, which depend on several factors including: (a) intrinsic factors (age of the hemorrhage, size, location, erythrocyte integrity and continued bleeding) (7); (b) biological factors (degree of oxygenation, blood-brain barrier integrity, local pH, hematocrit) (8); and (c) technical factors (magnetic field strength, homogeneity of the magnetic field, spin echo and gradient echo sequence types) (9).

Despite the precision of MRI for detecting intracranial hemorrhage the complex appearance of hemorrhage means that determining the underlying pathology responsible for the disruption to vascular integrity can be challenging. This is encountered most frequently when trying to establish the nature of a solitary, intra-axial, hemorrhagic, space occupying lesion; does the lesion represent a hematoma or a hemorrhagic neoplastic mass? In the case of a hematoma, options for clinical management include surgical correction and/or supportive treatment and monitoring for resolution of clinical signs (10). The prognosis for an intra-axial hematoma in humans is good and there is a strong association with return of normal functional independence reported (11). In dogs, a wide range of pathologies can result in benign intracranial hemorrhage and subsequent hematoma formation, including: vasculitis (12), hypertension (13), degenerative disease (14), vascular malformations (15), hemorrhagic transformation of ischemic stroke (16), *Angiostrongylus vasorum* infection (17, 18), and coagulopathies (19). However, in most cases of intracranial hematoma formation no underlying cause is found (20). Hemorrhage associated with intracranial neoplasia is suggestive of high-grade malignancy (21). Management options for neoplastic hemorrhagic lesions include radiotherapy with or without concurrent surgical intervention, or palliative treatment. Surgery may not be feasible and adverse effects can occur with both surgery and radiotherapy (22–24). The prognosis for dogs suffering from intracranial neoplasia is guarded to poor with a previous study reporting a median survival time of 1 month (25), and therefore, euthanasia is frequently opted for in these cases.

**Figure 1:**
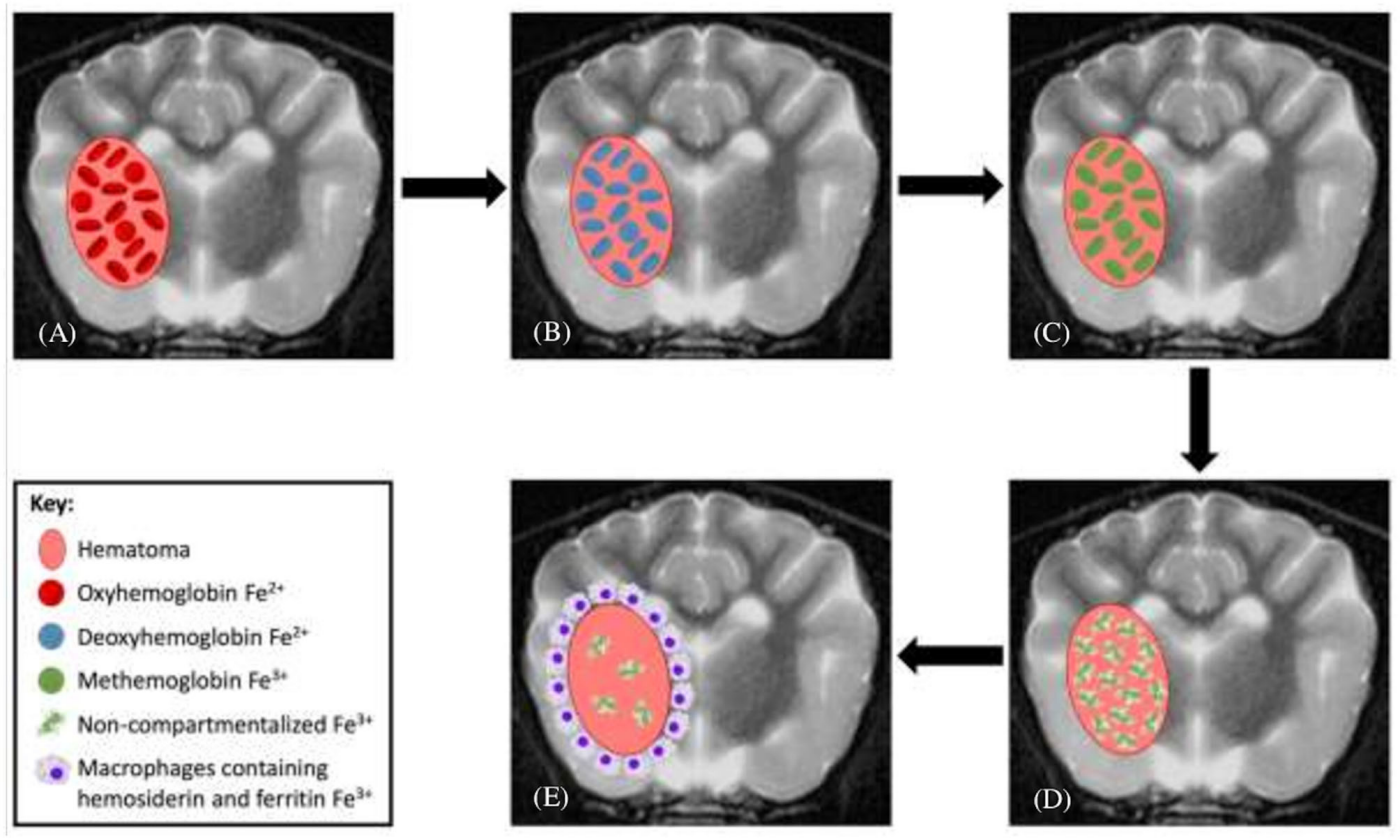
Evolution of intraparenchymal hemorrhage on magnetic resonance imaging. **(A)** In the earliest stage of acute hematomas, blood is still oxygenated within intact red blood cells (RBCs). **(B)** Rapid deoxygenation occurs, first at the periphery and then throughout the hematoma, but the RBCs remain intact. **(C)** As the lesion undergoes oxidation, the peripheral hemoglobin within intact RBCs forms methemoglobin. **(D)** This oxidation process and conversion to methemoglobin occur throughout the hematoma and subsequently the RBCs lyse. **(E)** As free methemoglobin is formed, hemosiderin and other iron storage forms are deposited within macrophages in the adjacent brain parenchyma. Eventually, the lesion contains no intact RBCs, and methemoglobin is resorbed or metabolized, leaving only a collapsed cleft lined by hemosiderin and ferritin without any notable central constituents.

In people, the MRI features that help to distinguish an intra-axial hematoma from malignant intralesional hemorrhage are well-documented (7, 26, 27). The MRI features of hematomas include: the temporal progression of the signal intensities during hematoma evolution occurs as expected with no delay in the transition between the blood-breakdown product phases (Table 1; Figure 1); hematomas are mostly solitary mass lesions which have a rounded/globular shape; during the acute and chronic phases, the lesions have a regular and complete ring of peripheral deoxyhemoglobin or hemosiderin, respectively (T1W and T2W hypointense ring); in the chronic stages, there is complete resolution of perilesional oedema and mass effect; and there is no evidence of abnormal non-hemorrhagic tissue within the lesion (26). MRI features of hemorrhagic malignancy directly oppose these changes and include: delayed temporal evolution of the hemorrhage; irregularly shaped mass lesion; diminished, irregular or absent hemosiderin deposition; pronounced or persistent oedema and mass effect; and notable signal heterogeneity, often with identifiable non-hemorrhagic tissue corresponding to tumor (7, 27) (These differences are summarized in Table 2).

**Table 1:** The temporal evolution of the MRI signal characteristics of intracranial hemorrhage reported in dogs compared to that observed in humans (3, 28).

	**Intensity on T1W images**		**Intensity on T2W images**		**Intensity on GRE images**	
**Time of appearance**	**Human**	**Dog**	**Human**	**Dog**	**Human**	**Dog**
≤ 24 h Hyperacute	Isointense	Hypointense	Hyperintense	Hypointense and hyperintense	Hyperintense	Hypointense
1 ≤ 2 days Acute	Isointense to hypointense	Hypointense	Hypointense	Hypointense and hyperintense	Hypointense	Hypointense
2 ≤ 7 days Early subacute	Hyperintense	Hyperintense	Hypointense	Hyperintense	Hypointense	Hyperintense
7 ≤ 14 days Late subacute	Hyperintense	Hyperintense	Hyperintense	Hyperintense	Hyperintense	Hyperintense
>14 days Chronic	Isointense to hypointense	Isointense to hypointense	Hypointense	Hypointense and hyperintense	Hypointense	Hypointense

**Table 2:** The MRI characteristics of intracranial intra-axial hematomas compared to those of intracranial intra-axial hemorrhagic neoplasia as reported in humans.

**Hematoma**	**Hemorrhagic neoplasm**
- Signal intensity pattern seen reflects the expected temporal stage of hemorrhage - Follows the expected evolution (resolution of mass effect and oedema) - Complete hypointense rim - Peripheric contrast enhancement and absence of solid portion	- Heterogeneity of the lesion with different stages of hemorrhage - Delayed evolution of the breakdown products of the hemoglobin (persistent mass effect and oedema) - Absent or incomplete hypointense rim

An accurate description of the MRI characteristics of intra-axial hematomas has yet to be reported in clinical canine patients. An experimental, non-clinical study reported that the temporal evolution of blood breakdown products in canine intracranial hematomas largely mirrors that seen in humans, but that red blood cell membrane degradation may happen at a faster rate with extracellular methemoglobin seen at an earlier time point (28) (Table 1). Further imaging characteristics of the hematoma lesions were not elaborated in this study.

The aim of this study was to describe the MRI features of canine intra-axial hematomas and to correlate those findings with the current understanding of hemorrhage evolution occurring in human brains (7, 26, 27). It was hypothesized that the MRI characteristics of canine intra-axial hematomas would mirror those observed in humans.

## Materials and Methods

### Study Population

In this retrospective multicenter descriptive study, medical records of three different referral institutions were reviewed to identify dogs that had intra-axial hematomas on MRI. Ethical approval was granted by the Ethical Review Committee of the University of Nottingham School of Veterinary Medicine and Science. Patient inclusion criteria were set as: (a) a complete brain MRI study was performed (including at least T2W, T1W, T2^*^ or SWI, and T1W post contrast sequences); (b) the MRI study confirmed a solitary hemorrhagic space-occupying intra-axial lesion, and subsequently; (c) there was histopathological confirmation of a benign hematoma (samples obtained from surgical intervention or necropsy), or a follow-up MRI that indicated resolution or marked improvement of the previous lesion with only supportive treatment, and/or the patients clinical signs resolved with only supportive treatment. In cases where diagnosis of an intra-axial hematoma relied upon clinical resolution alone, a sufficient follow-up period was defined as 3 months after first presentation. Patients were excluded if the MRI study or medical data was incomplete, if they had received cytotoxic therapy (e.g., chemotherapy or radiotherapy), if they had a suspected or known history of trauma, if they underwent repeat MRI and this revealed a worsening of the severity of the intracranial lesion or the lesion remained unchanged, and if insufficient clinical follow-up was available.

The following medical record data was recorded: age, breed, sex, presenting clinical signs and date of manifestation, initial neurological examination findings, date of first MRI, blood hematology and biochemistry results, results of infectious disease testing, medications prescribed, and date of re-examination(s) and clinical/neurological findings at re-examination.

### Image Acquisition and Image Review

MRI protocols and sequences varied between institutions, but all MRI examinations were completed with dogs under general anesthesia, using high-field-strength magnets: 1.5 Tesla (Hallmarq PetVet; Siemens Magnetom Sola; Toshiba Vantage Elan). Anesthetic protocols were varied and adapted to individual patients by the attending anesthetist. The MRI studies were reviewed by three observers independently [two ECVDI certified veterinary radiologists (AH and IC) and a ECVDI resident (JW)], followed by collective consensual evaluation. All available follow-up MRI studies were also evaluated. Reviewers were aware that all dogs had intra-axial hematomas. The following features were assessed on image evaluation:

- The location of the lesion, and the distribution of the lesion within either the gray or white matter.
- Lesion margination was characterized subjectively as well or poorly circumscribed.
- Lesion shape was defined as spherical, ellipsoidal, or irregular.
- The signal intensity of the lesion in each sequence was evaluated both at the lesions periphery and at its center and graded as hypointense, isointense, hyperintense, or mixed intensity, relative to cerebral gray matter on T1W, T2W, and T2^*^ or SWI.
- If there were distinct regional differences in the signal intensity between the periphery of a lesion and its center, then the diameter of the peripheral region was measured at its widest point.
- The degree of contrast enhancement was subjectively categorized (absent, faint or obvious). Furthermore, the pattern of enhancement was also noted (parenchymal homogeneous/heterogeneous, or peripheral enhancement).
- Perilesional edema was graded as absent, mild (present in the area immediately adjacent to the lesion), moderate (extending beyond the area immediately adjacent to the lesion) or marked (involving the entire hemisphere). The amount of edema was determined by the extent of abnormal hyperintensity on T2W images, which was T1W iso to hypointense, non-contrast enhancing, and exceeded the boundary of the hematoma.
- Any mass effect (shift of the falx cerebri, compression of the ventricular system, trans tentorial, and/or cerebellar herniation) was subjectively graded as mild, moderate, or severe.
- Finally each reviewer estimated the age of each lesion (<24 h, 1–2 days, 2–7 days, 7–14 days, or >14 days) based on its signal intensity pattern and how this correlated to temporal hemorrhage evolution according to previously published data in dogs (28) (Table 1).

All MRI examinations were assessed using a PACS workstation DICOM viewer (OsiriX Imaging Software, version 12.0MD, Berne, Switzerland). All measurements were performed collectively during group evaluation.

## Results

### Signalment and Clinical Findings

Ten dogs met the inclusion criteria. These included four Crossbreeds, two Staffordshire Bull Terriers, a Dachshund, a Border Collie, a Jack Russell Terrier, and a Yorkshire Terrier. Of these, five were neutered females and five were neutered males. The age of the dogs varied between 7 and 13 years (median age 9.8 years) and the weight varied between 5.4 and 25.6 kg (median weight 15.72 kg).

The presenting neurological signs for each patient are summarized in Table 3 alongside hematoma location and likely vascular territory. All dogs had an MRI performed within a week following recognition of clinical signs.

**Table 3:** The neurological signs in each dog at first presentation displayed alongside the location of each intra-axial hematoma as revealed by magnetic resonance imaging and the likely vascular territory involved.

**Case**	**Neurological signs**	**Location of hematoma**	**Vascular territory**
1	Compulsive pacing to the left, absent postural reactions in the right thoracic and pelvic limbs, and absent menace response in OD	Forebrain, affecting the white matter of the medial aspect of the left temporo-frontal lobe and internal capsule	Middle cerebral artery, lateral lenticulostriate arteries
2	Vacant demeanor, circling to the left, delayed right pelvic limb postural reactions, and frequent myoclonic partial seizures	Forebrain, affecting the white matter of the left frontal lobe (centrum semiovale)	Rostral cerebral artery
3	Left hemiparesis and reduced left menace response in OS	Forebrain, affecting the white matter of the right fronto-parietal lobe	Middle cerebral artery, lateral/medial lenticulostriate arteries
4	Generalized tonic-clonic seizures and tendency to move to the left	Forebrain, affecting the white matter of the left fronto-parietal lobe (radiation corpus callosum and substantia medullaris)	Rostral cerebral artery
5	Ataxia and circling to the right	Forebrain, right lateral aspect of the thalamus	Proximal deep perforating arteries
6	Left sided hemi-attention	Forebrain, white matter of the right occipital lobe	Middle cerebral artery
7	Generalized tonic-clonic seizures	Forebrain, white matter of the right frontal lobe (rostral internal capsule)	Middle cerebral artery, lateral lenticulostriate arteries
8	Generalized tonic-clonic seizures	Forebrain, white matter of the left parietal lobe	Middle cerebral artery, lateral/medial lenticulostriate arteries
9	Occasional left pleurothotonus otherwise neurologically normal	Forebrain, white matter of the left internal capsule to thalamus	Middle cerebral artery, lateral/medial lenticulostriate arteries
10	Vacant demeanor otherwise neurologically normal	Forebrain, white matter of the left frontal lobe (internal capsule)	Middle cerebral artery, lateral lenticulostriate arteries

Hematology and serum biochemistry results were available for all 10 patients. Hematologic abnormalities included mild neutrophilia (2/10), increased hemoglobin concentration (1/10) and mild anemia (1/10) and in all other cases the hematology results were normal. The only biochemical abnormalities identified were increased alanine aminotransferase (2/10), increased alkaline phosphatase (1/10), and hyperglycemia (1/10). All patients had clotting profiles within normal limits. All patients tested negative for *A.vasorum*. One patient was diagnosed with systemic hypertension on presentation (Case 9) via repetitive non-invasive indirect estimations of systolic blood pressure using a doppler devices. The hypertension was considered idiopathic in nature due to concurrent normal hematology, serum biochemistry, and urinalysis results. Aside from this patient, an underlying etiology responsible for the intracranial hemorrhage was not identified in the remaining cases. Records of blood pressure were not available in the remaining cases. Medical treatment prescribed to the patients included: prednisolone (5/10), fenbendazole (3/10), levetiracetam (4/10), and dexamethasone (2/10). All treatments were discontinued in all patients within 3 months of the initial presentation.

Histopathological confirmation of the diagnosis of a benign intra-axial hematoma was achieved in (2/10) of the patients. A diagnosis of a benign hematoma was made in (4/10) patients based on the results of a follow-up MRI study, which was also accompanied by neurological improvement. In the remaining four patients diagnosis was met by clinical follow-up alone, all these dogs were reported to have shown marked neurological improvement within 1 month after first presentation. On subsequent re-evaluations after 3 months all dogs were considered neurologically normal.

### MRI Findings

**Figure 2:**
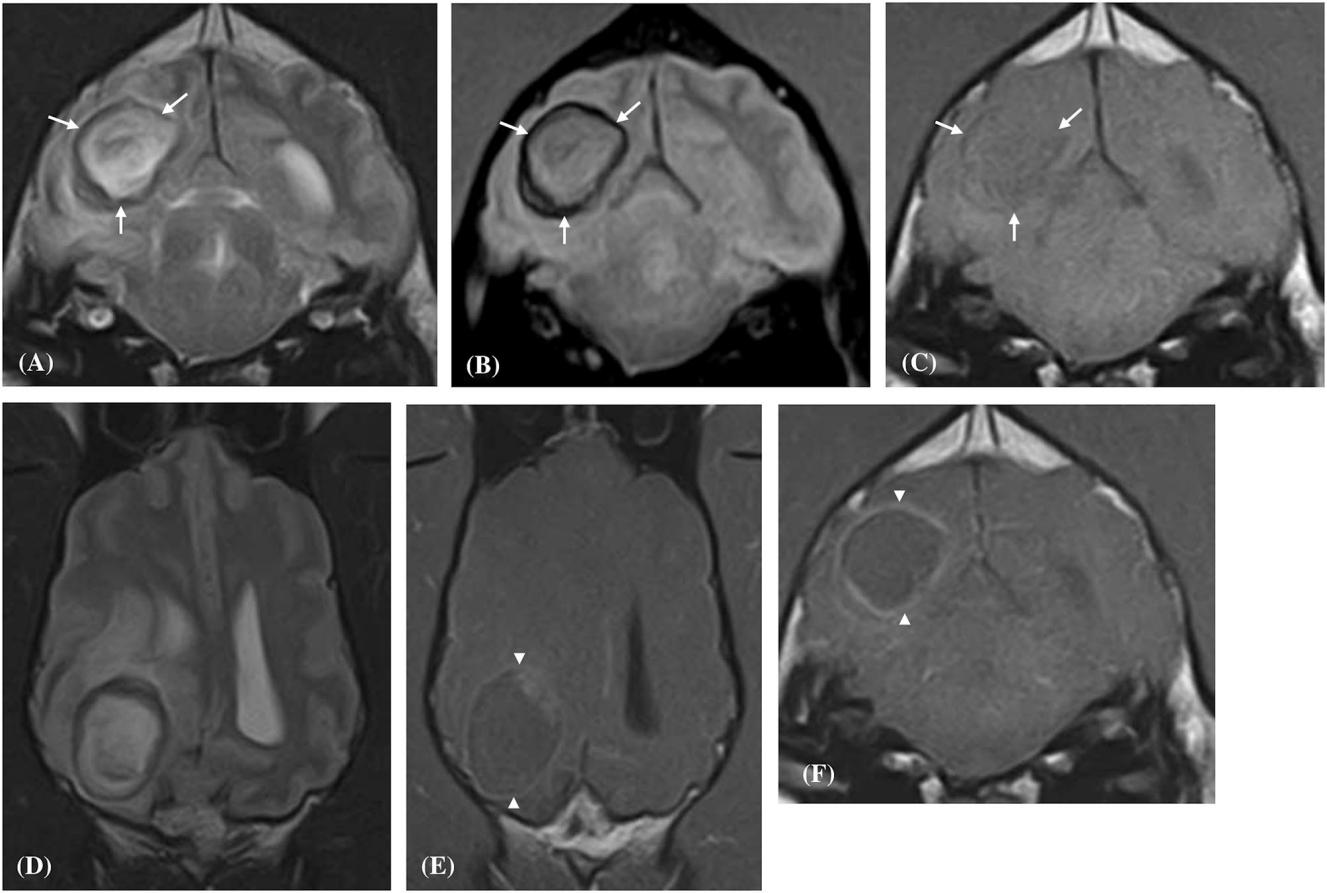
Transverse **(A–C,F)** and Dorsal **(D,E)** magnetic resonance images of a dog (Case 6) with a solitary well-defined intracranial intra-axial hematoma affecting the white matter of the right occipital lobe and associated perilesional oedema. The lesion displays a clear regional dichotomy with a hypointense thin, complete, circumferential peripheral border region (white arrows) and a large central predominately hyperintense region (relative to gray matter) on T2-weighted **(A,D)**, T2* gradient-echo **(B)** images. On pre-contrast T1-weighted images the peripheral border region is again hypointense **(C)** but is thinner relative to the T2-weighted images. Following contrast administration, the hematoma displays a thin peripheral enhancement pattern (white arrow heads) with no enhancement of the central portions of the lesion **(E,F)**.

**Figure 3:**
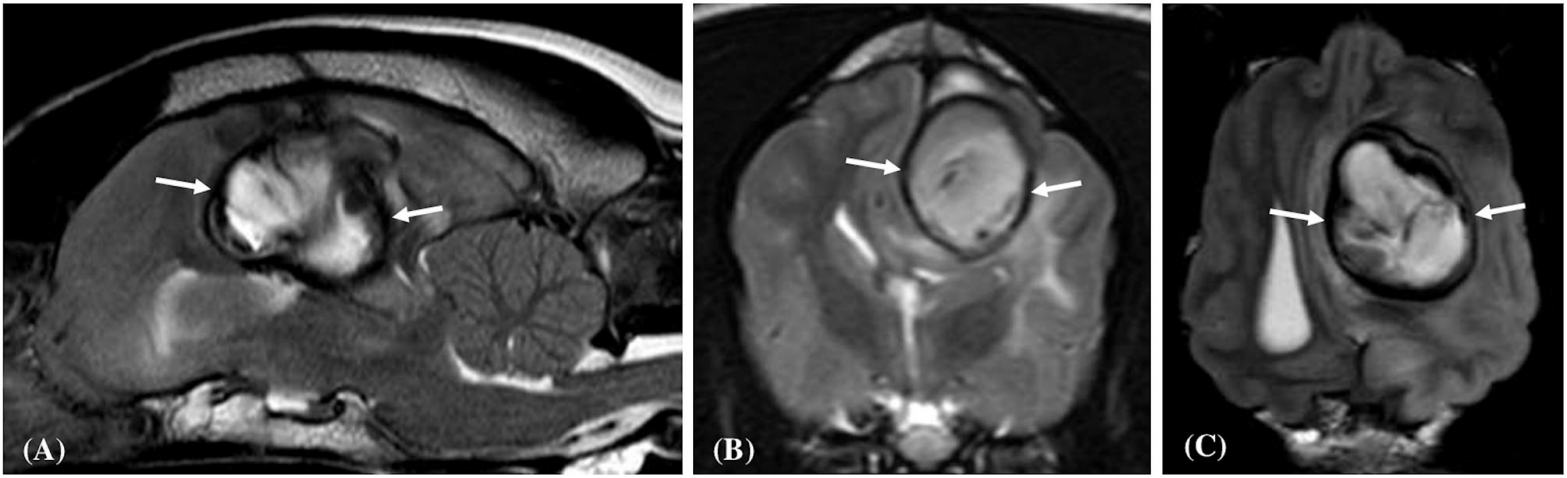
Left parasagittal **(A)**, transverse **(B)**, and dorsal **(C)** plane T2- weighted images of a dog (Case 8) with a large well-defined intracranial intra-axial hematoma affecting the white matter of the left parietal lobe. Note the circumferential nature of the thin hypointense rim (white arrows) in all three planes. Additionally, the lesion induces a marked mass effect resulting in transtentorial herniation, subfalcine herniation and compression of the left lateral ventricle.

In all 10 dogs the hematoma lesion was located within the forebrain and in 9/10 the lesion was localized to the white matter. The most frequently involved forebrain substructure was the frontal lobe (6/10, 60%), followed by the parietal lobe (3/10, 30%), the thalamus (2/10, 20%), and then single cases of occipital and temporal lobe involvement (Table 3). All 10 hematoma lesions were classified as well-marginated. Eight of the hematoma lesions had an ellipsoidal shape, and the remaining two lesions were classified as spherical. When evaluating the entirety of each hematoma, every hematoma was classified as having a heterogenous signal intensity pattern in T2W and T1W sequences. This heterogeneity was attributable to two distinct regions of signal intensity within all 10 hematomas: a thin peripheral border region and a large central region (Figure 2). The peripheral region was hypointense in all 10 hematomas on T2W and gradient echo sequences. The thickness of this peripheral region on T2W sequences was categorized as 1–5 mm in all cases. Additionally, the peripheral region was T1W hypointense in nine cases and of mixed intensity (isointense to hyperintense) in a single case. The thickness of the peripheral region on T1W images was less than that measured on the T2W sequences in all 10 cases (Figure 2). This hypointense peripheral region was complete in its circumference in all 10 hematomas with no break in its continuity (Figure 3). On T1W sequences the central region was of mixed intensity in (7/10) [hypointense to isointense (4/7), isointense to hyperintense (3/7)], hyperintense in (2/10), and isointense in (1/10) of the hematoma cases. On T2W sequences the central region was hyperintense in (5/10), of mixed intensity in (4/10) [hypointense to hyperintense (2/4), and isointense to hyperintense (2/4)], and hypointense in (1/10) of hematoma cases. On gradient echo sequences the central region was hypointense to hyperintense in (7/10) and hyperintense in (3/10) of the hematoma lesions. The signal intensities of the hematomas across the different MRI sequences are summarized in Table 4. Based on these intensity patterns the predominant blood breakdown product within the peripheral region of the hematoma was interpreted to be ferratin and hemosiderin in (9/10) of cases and in a single case it was thought to be intracellular methemoglobin. The predominant blood breakdown product within the central region of the hematoma was interpreted to be extracellular methemoglobin in (8/10) cases, and intracellular methemoglobin in two cases. All hematomas were classed as being 2–7 days old based on their signal intensity patterns and this reflected their clinical time course.

**Table 4:** Displays the signal intensities of the peripheral and central regions of the solitary intra-axial hematoma lesions in each case on T1 weighted, T2 weighted and gradient echo sequences.

	**Periphery of the hematoma lesion**			**Center of the hematoma lesion**		
**Case number**	**T1 weighted**	**T2 weighted**	**Gradient echo**	**T1 weighted**	**T2 weighted**	**Gradient echo**
1	Hypointense	Hypointense	Hypointense	Isointense–hypointense	Hypointense–hyperintense	Hypointense–hyperintense
2	Hypointense	Hypointense	Hypointense	Isointense–hypointense	Hyperintense	Hyperintense
3	Hypointense	Hypointense	Hypointense	Isointense–hypointense	Hypointense	Hypointense–hyperintense
4	Isointense–hyperintense	Hypointense	Hypointense	Isointense	Isointense–hyperintense	Hypointense
5	Hypointense	Hypointense	Hypointense	Hyperintense	Hyperintense	Hypointense–hyperintense
6	Hypointense	Hypointense	Hypointense	Isointense–hyperintense	Hyperintense	Hyperintense
7	Hypointense	Hypointense	Hypointense	Isointense–hypointense	Hypointense–hyperintense	Hypointense–hyperintense
8	Hypointense	Hypointense	Hypointense	Isointense–hyperintense	Hyperintense	Hypointense–hyperintense
9	Hypointense	Hypointense	Hypointense	Isointense–hyperintense	Isointense–hyperintense	Hypointense–hyperintense
10	Hypointense	Hypointense	Hypointense	Hyperintense	Hyperintense	Hyperintense

**Figure 4:**
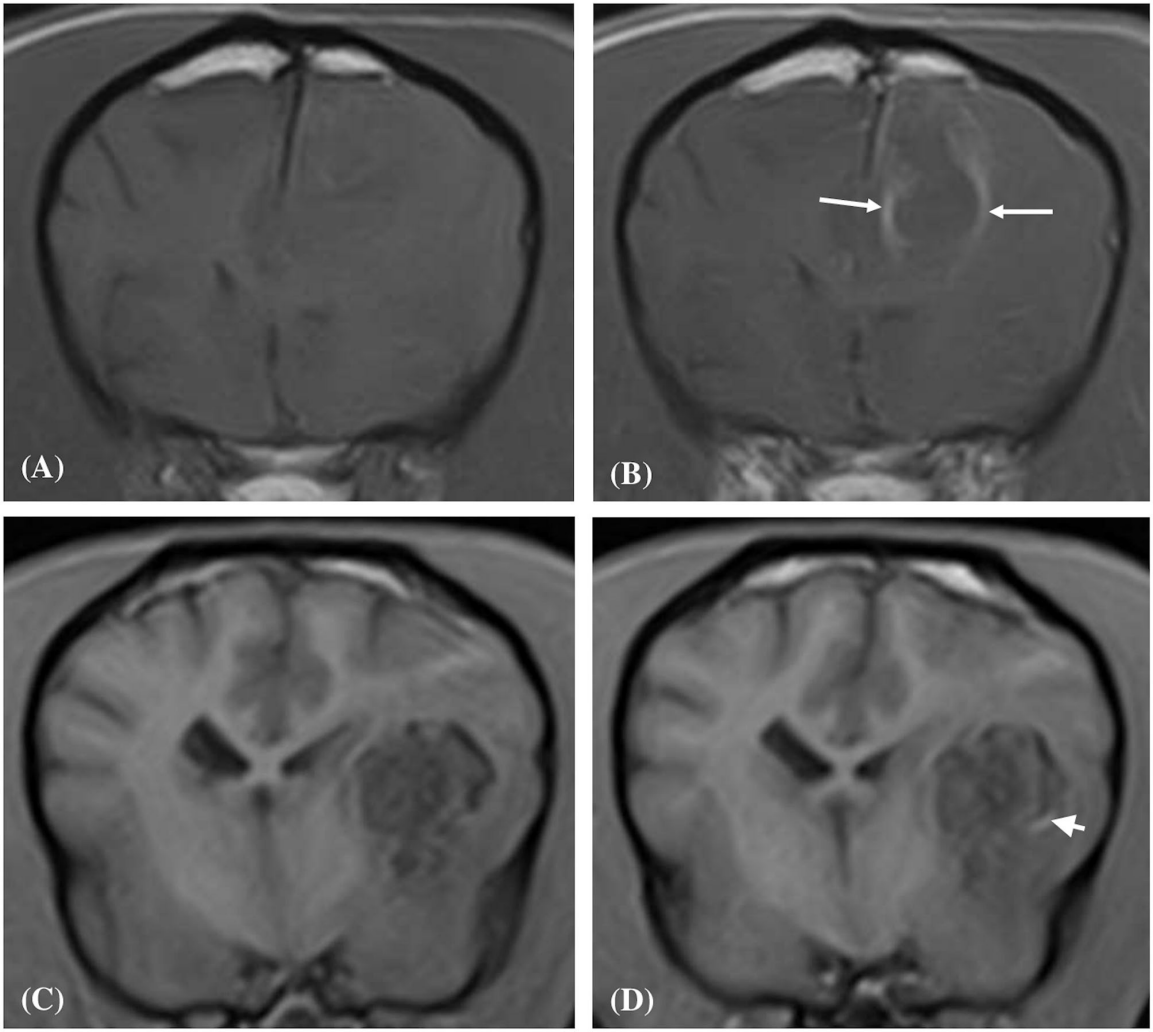
Transverse plane T1-weighted **(A)**, and T1-weighted post contrast **(B)**, images of a dog (Case 4) with an intracranial intra-axial hematoma affecting the white matter of the left fronto-parietal lobe. The lesion displays an obvious peripheral contrast enhancement pattern (long white arrows) **(B)**. Transverse plane T1-weighted **(C)**, and T1-weighted post contrast **(D)** images of dog (Case 1) with a hematoma lesion affecting the white matter of the medial aspect of the left temporo-frontal lobe and internal capsule. The lesion displays a faint and focal area of peripheral contrast enhancement (short white arrow). Neither lesions post contrast **(B,D)** show enhancement of their central regions.

Contrast enhancement was evident in (6/10) hematomas and absent in (4/10) cases. When present the enhancement was always present at the periphery of the hematoma (Figures 2, 4). Enhancement was deemed obvious in (4/6) and faint in (2/6) cases. There were no regions of central enhancement within any of the hematoma lesions. Perilesional oedema was classified as moderate in (6/10) and mild in (4/6) hematomas. A mass effect was associated with all hematoma lesions. The mass effect was deemed moderate in (6/10), mild (3/10) and marked in (1/10) of the hematoma cases. A lateral shift of the falx cerebri was noted in (7/10) cases, compression of the lateral ventricles in (4/10) cases, and caudal transtentorial herniation was observed in a single case.

**Figure 5:**
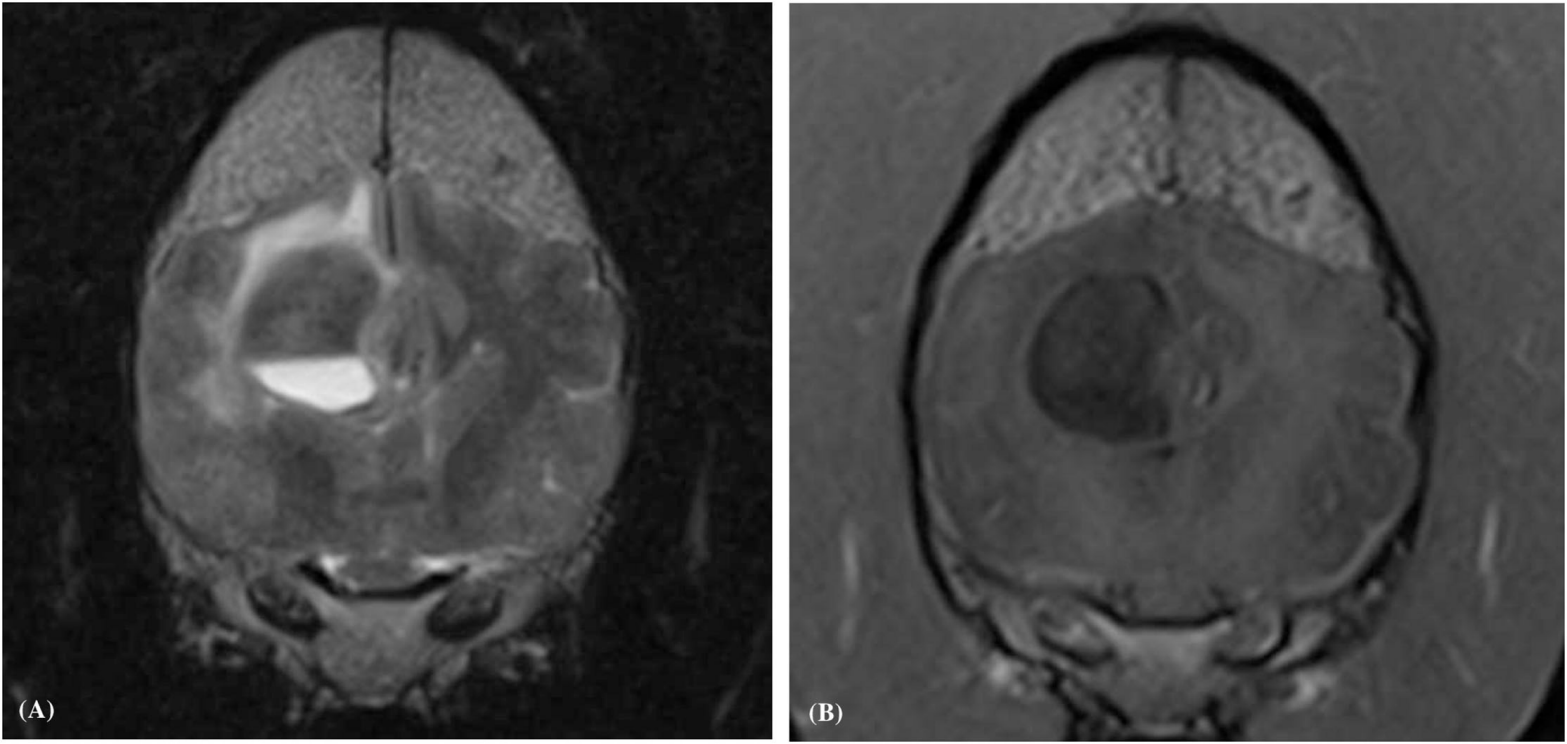
Transverse plane T2-weighted **(A)**, and T1-weighted post contrast **(B)**, images of a dog (Case 3) with an intracranial intra-axial hematoma affecting the white matter of the right fronto-parietal lobe. In this section of the lesion there is a well-defined fluid-fluid lesion. The dependent and ventral region is T2-weighted hyperintense and T1-weighted hypointense, and the dorsal region was T2-weighted hypointense and T1-weighted isointense relative to gray matter. This imaging feature was only observed in two of the hematoma lesions.

Fluid-fluid level lesions were evident within the central portions of the hematomas in Case 3 and 4. In Case 3 this feature manifested as the dependent and ventral region being T2W hyperintense and T1W hypointense, and the dorsal region being T2W hypointense and T1W isointense (Figure 5). In Case 4 this feature was completely T1W isointense and was composed of three regions on the T2W images; a hypointense to isointense region dorsally, a hyperintense region at its mid aspect, and a hypointense region ventrally.

**Figure 6:**
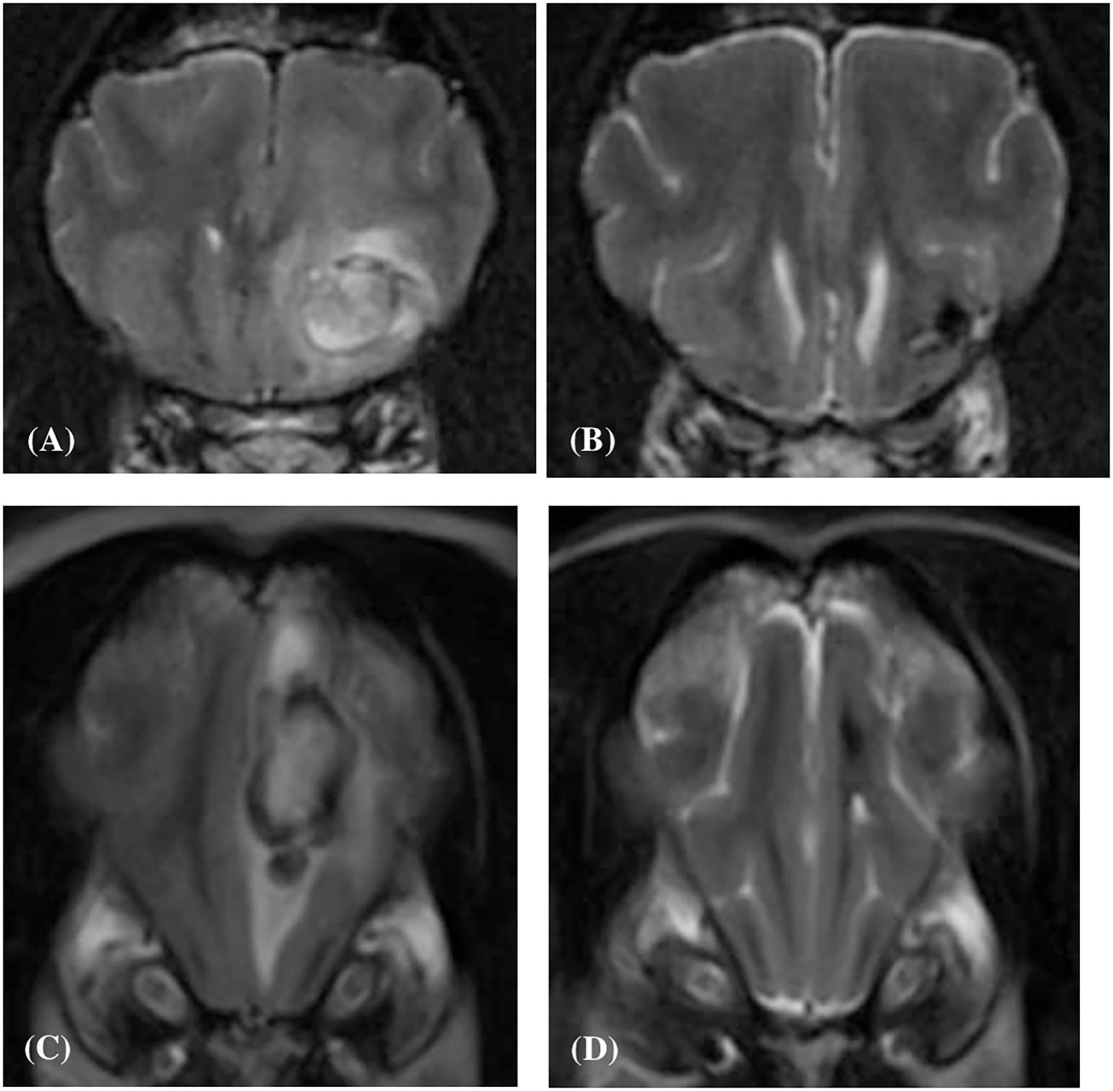
Transverse T2-weighted image **(A)** of a dog (Case 10) with an intracranial intra-axial hematoma affecting white matter of the left frontal lobe at first presentation. Transverse T2-weighted image **(B)** of the same dog as displayed in **(A)** at the same location but 83 days later. Transverse T2-weighted image **(C)** of a dog (Case 2) with an intracranial intra-axial hematoma affecting the white matter of the left frontal lobe (centrum semiovale) at first presentation. Transverse T2-weighted image **(D)** of the same dog as displayed in **(C)** at the same location but 207 days later. In both the follow up images **(B,D)** the lesions have reduced dramatically in volume, are uniformly hypointense and perilesional oedema has resolved. This is illustrative of normal intra-axial intracranial hematoma contraction and maturation.

Four out of the ten dogs had repeat MRI studies; case 2, case 4, case 8 and case 10 at 207, 32, 46, and 83 days following their initial MRI study, respectively (median value; 64.5 days). On evaluation of these follow-up studies, it was observed that in all four cases the lesions had markedly reduced in volume, were hypointense in all sequences, and both perilesional edema and contrast enhancement were absent (Figure 6).

## Discussion

This study demonstrates that in the period of 2–7 days post hemorrhage, intra-axial hematomas most commonly appear as ordered dichotomous lesions on MRI images consisting of a complete T2W and T2^*^W hypointense peripheral border region that is also T1W hypointense but thinner, and a large predominantly T2W hyperintense central region. Additionally, intra-axial hematomas were shown to have mild to moderate peripheral contrast enhancement with no central enhancement. Furthermore, hematomas were frequently associated with mild to moderate mass effect and mild to moderate perilesional edema. These findings largely reflect the MRI characteristics of intra-axial hematomas observed in humans.

The fact that all hematoma lesions in this study demonstrated a uniform regional duality consisting of a complete thin peripheral region and a large central region is in direct agreement with human studies (26, 29). This dichotomy and central/peripheral divide are due to the difference in the blood breakdown products present in these regions, and consequently leads to the differences in signal intensity observed. The reason for the difference in blood breakdown products between these two regions is that the temporal evolution of these products is faster at the lesion periphery (26). This is due to very rapid deoxygenation of blood within the hematoma at the peripheral blood–tissue interface (26). Animal studies have demonstrated that ferritin or hemosiderin are present in the peripheral rim region of a hematoma <24 h following hemorrhage (hyperacute phase) (29). In human studies it has been demonstrated that the transition of blood breakdown products within an intra-axial hematoma over time occurs from the periphery toward the center of the lesion (26, 30). In benign intra-axial hematomas, this centripetal hematoma maturation process occurs in an ordered manner leading to the expected signal intensity changes seen at different time points on MRI (26). The uniform dichotomous pattern of the intra-axial hematomas demonstrated in this study is important as it is distinct from the markedly heterogenous appearance of hemorrhagic malignant neoplasms seen in human studies (7, 27). Due to the progressive nature of tumors there can be fresh hemorrhagic foci occurring at different time points and at irregular locations within a lesion. Typically, this means that neoplastic lesions do not present as lesions made up of two distinct uniform regions of different signal intensity, but rather as lesions with multiple irregularly distributed and shaped regions of different signal intensity (7, 27). It is proposed that benign hematomas have their dual region pattern due to the fact they tend to be generated from a solitary hemorrhagic event (26).

When considering the predominant blood breakdown products within the hematoma lesion it is important to bear in mind the peripheral to central nature of hematoma maturation. The “oldest” or most advanced breakdown products will be present at the periphery of the lesion. When considering the likely constituents of the peripheral border in the hematoma lesions in our study, this factor was considered alongside the hypointense signal of this region in all sequences and the pattern of signal intensity within the central region that showed loss of the magnetic susceptibility effect (indicating the presence of extracellular methemoglobin). Consequently, the peripheral border had to be ferritin and hemosiderin and not a less advanced product like deoxyhemoglobin that would have also satisfied the signal intensity pattern seen.

Another key differentiating feature of intra-axial hematomas seen in this study is the complete uninterrupted nature of the T2 & T2^*^ weighted hypointense peripheral region noted in all 10 cases. This is in agreement with previous human literature which also found that the presence of a complete peripheral hypointense rim in a hemorrhagic lesion was associated with benign intra-axial hematomas (26). Conversely, it has been reported that the disruption or absence of the hypointense rim in a hemorrhagic lesion is associated with hemorrhagic neoplasms (7, 27). This may be explained by blood–brain barrier disruption known to occur with intracranial malignancies, which results in more efficient removal of iron storage products (7). A thinner T1W hypointense complete peripheral border was also observed in 9/10 cases (in a single case the rim was present and complete but considered isointense to hyperintense). However, this had to be assessed in three orthogonal planes in some cases as partial volume averaging artifact resulted in apparent disruption of the peripheral border if only assessed on a single plane.

A large proportion of the hematoma lesions in this study showed contrast enhancement and in each case a faint to obvious peripheral pattern enhancement with no evidence of central intrahematoma enhancement was seen. In a computed tomography study involving canine patients with expermentally induced intracerebral hematomas in the region of the basal ganglia, similar peripheral ring enhancement was also observed (31). On histopathological examination, the area of enhancement coincided exactly with new connective tissue formed from argentophil fiber layers and the endothelium of newly formed capillaries; it was therefore hypothesized that the enhancement was as a result of increased permeability due to these newly formed capillaries (31). It has been reported that MRI contrast enhancement of benign hematomas does not occur until the subacute stage, whereas in hemorrhagic tumors it can occur at earlier time points (7, 26). Intralesional enhancement at any stage post hemorrhage and/or irregular or nodular enhancement outside the area of the hemorrhage has been strongly associated with an underlying neoplastic processes (7, 27).

A follow-up MRI study was performed in four cases and in all of them the hematoma lesion had reduced in size, was hypointense in all sequences, and both perilesional oedema and contrast enhancement were absent. This is in agreement with what has been reported in the human literature (26). Effectively, the thin T1W and T2W hypointense rim of iron storage forms ferritin and hemosiderin begin to thicken in a concentric centripetal fashion as the hematoma resolves in conjuncture with contraction of the hematoma in on itself, until these forms predominate throughout the lesion's entirety. An area of marked hypointensity on T2-weighted images and particularly on gradient echo images can remain at the site of an old hemorrhage indefinitely, which represents a residual cleft of hemosiderin and ferritin (26, 28). Importantly, this process of peripheral hemosiderin evolution is absent or delayed in cases of intratumoral hemorrhage (7, 27). This overall delay in hemorrhage evolution in malignant lesions has been postulated to be caused by profound intratumoral hypoxia, which has been documented in human neoplasms (32).

Mass effect was observed with all the intra-axial hematoma lesions and varied from mild to marked in its severity, with a moderate mass effect most frequently observed. Additionally, the size of the hematomas was highly variable with some being of considerable volume. This highlights that the presence of a large hemorrhagic intra-axial space occupying lesion cannot be automatically categorized as a neoplastic process.

This study also supports previous published data suggesting that the normal temporal evolution of intracranial hematomas is more rapid in dogs than in man, with red blood cell lysis occurring at in the 4–7 day period (early subacute phase) rather than the 7–14 day period (late subacute phase) following the hemorrhage (28). Compartmentalisation of iron within the RBC membrane causes inhomogeneity in the magnetic field (the “magnetic susceptibility effect”) and thus shortens T2 relaxation time without affecting T1 relaxation time. When the RBC membrane degrades the susceptibility effect disappears, the accelerated T2 relaxation is lost, and the signal on T2W images brightens (33). All the dogs in our study were imaged within <7 days of the onset of neurological signs/hemorrhage and a large proportion demonstrated loss of the magnetic susceptibility effect and hyperintensity within the central portion of the hematoma on gradient echo and T2W images, correlating to the presence of extracellular methemoglobin. This is an important concept when it comes to interpreting the age of an intra-cranial hemorrhage, and the use of the well-established timings for the appearance of these different breakdown products following hemorrhage cannot be rigidly applied. This is true in two respects; the rate of blood breakdown product evolution in dogs is different from that of man, and more than one form of blood breakdown product will be present within a lesion at any one time with products traditionally thought of as being seen in the more chronic stages being present much earlier in the hemorrhagic process.

Two hematomas had regions where a fluid-fluid interface was evident. This feature in intracranial hemorrhage has been associated with coagulopathic disorders (34). However, the clotting profiles of both affected dogs were normal. In hemorrhagic intracranial neoplasms, fluid-fluid interfaces can also be present as cystic or necrotic regions containing hemorrhage debris or hemorrhagic fluid levels (7). Both of these dogs were neurologically normal following a 1-month period of conservative treatment, and one of the dogs (Case 4) had a follow-up MRI study which showed normal hematoma evolution and contraction.

The underlying etiology for the development of the intracranial hematoma was not established in 9/10 cases. This is not uncommon with a previous study observing that 32 of 43 dogs (74%) with a solitary intracranial hematoma had no concurrent underlying medical condition and the underlying etiology could not be established (20). The underlying etiologies that result in intracranial hemorrhage and subsequent hematoma formation can be classified as either primary or secondary. Primary causes include hypertension and ceroid amyloid angiopathy which result in spontaneous rupture of the vasculature, but the reported incidence of intracranial hemorrhage caused by these pathologies is low (14, 35, 36). Secondary causes (excluding neoplasia) include thrombosis, infection, trauma, cerebral vascular malformation and coagulopathy (12, 15, 17–19).

The principal limitation of this study is the small number of cases where gold-standard histopathological confirmation of a hematoma was available. Nonetheless, resolution of the clinical and neurological signs, long-term clinical follow-up, and a repeat MRI study (where available) for the remaining cases provides a reasonable assertion that these lesions were attributable to simple hematomas rather than hemorrhagic malignancies. The subjective and qualitative nature of assessment of multiple factors on the MRI studies (including signal intensities, lesion conformation, and grading of perilesional oedema and mass effect) may have also led to inaccuracies. However, the individual and collective group assessments of the MRI findings may have mitigated these to a degree. Additionally, the study was limited by the low numbers present in the sample population, but this reflects the uncommon occurrence and/or identification of solitary benign intra-axial hematomas in the clinical setting. Serial monitoring of the hematoma lesions with MRI over time would have been rewarding to document the characteristics of more chronic phases of hematoma evolution, but this was not viable in the clinical setting. Finally, it must always be kept in mind that the imaging characteristics of hemorrhage are linked to magnetic field strength with higher magnetic field strengths enhancing susceptibility effects and related signal loss. Consequently, this changes the appearance of hematomas at 1.5 T relative to lower magnetic field strengths because more profound hypointensity will be shown in the presence of deoxyhaemoglobin, hemosiderin, or other compartmentalized paramagnetic material (37).

In conclusion, this study suggests that a benign intra-axial hematoma should be considered as a primary differential when a solitary intra-axial hemorrhagic lesion with a complete thin hypointense peripheral rim, a variable intensity central region, and a peripheral enhancement pattern is identified on MRI. Accurately differentiating between benign intra-axial hematomas and hemorrhagic tumors using MRI is challenging and can be stressful for all those involved in the clinical decision-making process, particularly given that euthanasia or an expensive treatment could be chosen if a malignancy is suspected. This is further compounded by the fact that a definitive diagnosis is seldom achieved for such lesions antemortem. In this scenario, depending on the patient's clinical status and severity of the neurological signs, the authors propose that it would be prudent to monitor the patient for further improvement or deterioration combined with follow-up advanced imaging, if feasible. Further studies could also be conducted evaluating the potential inverse MRI characteristics of solitary intra-axial hemorrhagic neoplasms in dogs.

## Data Availability Statement

The original contributions presented in the study are included in the article/supplementary material, further inquiries can be directed to the corresponding author/s.

## Ethics Statement

The animal study was reviewed and approved by Ethical Review Committee of the University of Nottingham School of Veterinary Medicine and Science. Written informed consent was obtained from the owners for the participation of their animals in this study.

## Author Contributions

IC conceived of the presented idea. JW, IC, and AH performed the image evaluation. JW wrote the manuscript. All authors contributed cases and discussed the results and contributed to the final manuscript.

## Conflict of Interest

The authors declare that the research was conducted in the absence of any commercial or financial relationships that could be construed as a potential conflict of interest.

## Publisher's Note

All claims expressed in this article are solely those of the authors and do not necessarily represent those of their affiliated organizations, or those of the publisher, the editors and the reviewers. Any product that may be evaluated in this article, or claim that may be made by its manufacturer, is not guaranteed or endorsed by the publisher.
